# Behavior and Characteristics of Sap-Feeding North Island kākā (*Nestor meridionalis septentrionalis*) in Wellington, New Zealand

**DOI:** 10.3390/ani3030830

**Published:** 2013-08-16

**Authors:** Kerry E. Charles, Wayne L. Linklater

**Affiliations:** Centre for Biodiversity and Restoration Ecology, School of Biological Sciences, Victoria University of Wellington, P.O. Box 600, Wellington, New Zealand; E-Mails: kerryecharles@gmail.com

**Keywords:** sap-feeding, foraging, bark stripping, human-wildlife conflict, wildlife damage, urban wildlife

## Abstract

**Simple Summary:**

Understanding the behavior of problem animal species assists in understanding and mitigating problems caused by wildlife in urban landscapes. The kākā, a threatened New Zealand native parrot, causes damage to trees while feeding on sap. Through observations of sap foraging kākā in Wellington City, this study builds on the limited knowledge of sap feeding and tests hypotheses about the age and sex of sap feeding birds. We found that sap feeding likely occurs in both sexes and across age groups, and that sap feeding birds also utilize supplementary food. This study suggests that sap is an important food source for kākā and that further provision of supplementary food is unlikely to reduce sap feeding and associated tree damage.

**Abstract:**

The North Island kākā (*Nestor meridionalis septentrionalis*), a threatened New Zealand native parrot, was successfully reintroduced to an urban sanctuary in Wellington, New Zealand. Conflict has recently begun to emerge with Wellington City residents due to tree damage caused by kākā sap foraging. Little is known about sap foraging behavior of kākā, and this study aimed to gain a greater understanding of this behavior, and to test hypotheses that sap feeding is predominantly a female activity and that one technique, forming transverse gouges through bark, may be restricted to adult kākā. We used instantaneous scan sampling to record the behavior of kākā during 25 60–100 minute observation periods at Anderson Park, Wellington Botanic Garden, and during 13 opportunistic observations of sap feeding kākā in Wellington City. Forty-one observations of sap feeding were made of 21 individually-identified birds. Sap feeding birds were predominantly young and, based on estimated sex, females were no more likely to sap feed than males (exact binomial test *p* = 0.868). Twenty of the 21 identified sap feeding kākā utilized supplementary feeding stations at Zealandia-Karori Wildlife Sanctuary. Kākā were observed defending sap feeding sites from tui (*Prosthemadera novaeseelandiae*) and conspecifics. Sap appears to be an important resource for kākā across sexes and life stages, and provision of supplementary food is unlikely to reduce sap feeding and tree damage in Wellington City.

## 1. Introduction

In recent decades, the success of urban wildlife conservation and the restoration of urban wildlife habitats have led to growing animal populations in cities [1,2,3] and an increase in urban human-wildlife conflict [4,5]. The urban landscape can have dramatic effects on a species, altering behavior, ecology, population dynamics and habitat utilization [6,7]. Hence, urban-specific knowledge of the ecology and behavior of conflict-causing species is needed in order to manage wildlife and conflict in an urban landscape [8]. 

The kākā (*Nestor meridionalis*: Nestoridae), a threatened parrot endemic to New Zealand, feeds on sap by removing bark from a range of tree species. Although kākā have a diverse diet, sap is an important seasonal food source and considerable time may be spent foraging for sap [9,10]. 

The only population of kākā in an urban landscape is found in Wellington City. Kākā were reintroduced to the urban Zealandia-Karori Wildlife Sanctuary (hereafter KWS, 41.290°S 174.753°E) [11] in 2002 and the population established and bred successfully. Kākā are free to disperse beyond the sanctuary’s mammal predator exclusion fence and now frequent the cities’ inner suburbs [12]. Conflict with Wellington City residents is beginning to emerge predominantly due to tree damage resulting from sap feeding. Damage from sap feeding can impact on tree health and structural integrity, and, in Wellington, has necessitated branch, crown and entire tree removal for residents’ safety [13]. Bark damage has been observed on a wide range of native and exotic tree species in Wellington City, including trees with considerable amenity and historical value [13]. 

Knowledge about kākā sap feeding behavior is limited. Previous descriptions and studies of sap feeding in the literature have been limited by small sample sizes, with a maximum of four sap-feeding individuals identified [9] and little demographic information for observed birds has been available. Sap feeding has been fortuitously observed in the native forests of south Westland [10] and Mt Bruce Reserve, Wairarapa [14] during other studies of kākā behavior. The only study to focus on sap feeding investigated the behavior and resulting tree damage in exotic plantations and adjoining native forest in Whirinaki, Bay of Plenty region [9]. Six birds were tracked, four were observed sap feeding, and sap foraging was found to comprise 24% of all foraging observations. 

It has been suggested that sap feeding may be predominantly a female behavior [9,14,15] and that one sap foraging technique, transverse gouging through bark, may be restricted to older kākā [14]. This has led to the hypothesis that sap may be an important, high-energy food source for females prior to breeding [9]. It has also been speculated that female kākā may use hormonal indicators or increased nutrient content in sap to predict mast seed production and hence trigger the onset of breeding [15,16]. These hypotheses are largely speculative due to the paucity of research. 

Understanding sap feeding behavior and the characteristics of sap feeding birds has important implications for predicting future tree damage in Wellington City, with demographic change and population growth likely in the Wellington kākā population. It is crucial to understand sap feeding behavior in order to develop effective strategies to mitigate the emerging conflict caused by tree damage. For example, Innes [17] suggested that provision of supplementary high-energy foods may reduce sap foraging damage in plantation forests, however this relies on the untested hypothesis that sap feeding occurs as a response to energy needs.

The aims of this study are to, (a) describe and quantify sap feeding behavior in Wellington City, and to, (b) test hypotheses that sap feeding is predominantly restricted to female kākā [9,14,15] and that more specialized horizontal gouging methods of extracting sap are restricted to older birds [14]. This will provide a greater understanding of kākā sap feeding and is a necessary first step for the management of conflict. 

## 2. Methods

### 2.1. Study Population

North Island kākā (*Nestor meridionalis septentrionalis*) were reintroduced to Wellington City in August 2002 with the release of six captive-reared kākā to KWS [11]. Subsequent additional releases and considerable breeding success have led to a current estimated population in the Wellington area of approximately 180–250 birds [18]. The age of chicks that fledge from artificial nest boxes provided within KWS is known and they are fitted with individually-identifiable colored leg bands. Due to recent reintroduction and breeding success there is a higher proportion of young birds than adults in the population. The sex ratio for males and females of confirmed sex (n = 97) does not differ significantly from 0.5 [18], suggesting that there is a relatively balanced sex ratio in the Wellington City population as a whole. Within KWS, supplementary food, comprising sugar-water and commercially-prepared parrot pellets and nectivore mix, is provided year-round [12]. 

### 2.2. Behavioral Observations

**Figure 1:**
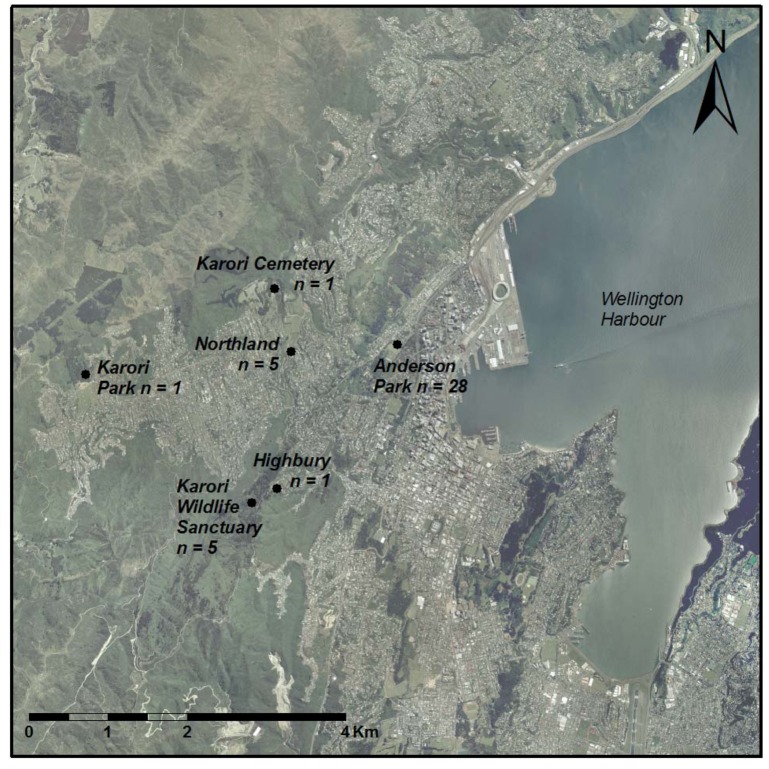
Locations of the six sites in Wellington City where kākā were observed sap feeding between November 2011 and May 2013, showing the number of times sap feeding was observed at the site.

Observations of kākā engaged in sap feeding were made during systematic behavioral sampling in the Anderson Park area of the Wellington Botanic Garden (−41.279°S, 174.770°E), and opportunistically on public and private property throughout Wellington between November 2011 and May 2013 (Figure 1). 

Anderson Park was selected as the site for systematic behavioral sampling as preliminary observations of kākā and tree damage indicated that this area was frequented by kākā. Observations began approximately one hour before sunset and ended when poor light prevented accurate observation. Observations were made 4–5 days per week over a six week period between mid-August and late-September 2012. This time of year was chosen as previous studies had shown this was a time of high sap feeding and anecdotal observations indicated this may also be true in Wellington City. 

Due to the rarity of observing sap feeding and the difficulty of predicting where and when it would occur, opportunistic focal behavior sampling was also adopted. This ensured as many observations as possible of sap feeding behavior could be included in the study. Opportunistic observations took place in public parks, gardens and bush fragments, including KWS, and on private residential properties throughout Wellington City.

During both systematic and opportunistic sampling, the locations of kākā were identified by kākā vocalizations or searching visually, and whenever a kākā was observed removing bark from a tree it became the focal bird and sampling began. An instantaneous sampling method [19] was used whereby the behavior of the focal bird was recorded at 1-minute intervals. Sampling continued until the bird was no longer in view (*i.e.*, the bird moved away or it became too dark to clearly see behavior) or until sap feeding ended and other behaviors were observed for at least 10 minutes. The leg band combinations of the focal bird and any nearby conspecifics were identified. The time and date, location, weather and tree species being fed on was also recorded. Other behaviors were noted, including conspecific and heterospecific interactions and vocalization.

The presence of an observer was assumed to have little effect on focal bird behavior as human presence and movement were common in the observation area and birds did not appear to be disturbed by the observer or other humans during observations. Three observations of sap feeding were supplied by staff and volunteers of Karori Sanctuary Trust and were judged to be reliable descriptions from experienced observers of kākā. All other observations were made by KEC.

### 2.3. Analysis

Observations were collated and analyzed to produce a description of sap feeding behavior. Records obtained from Karori Sanctuary Trust were used to determine the age (season fledged) and sex of identified birds [18]. Sex categorization was based on behavioral observation during breeding or predicted using a model based on discriminant function analysis of nestling body measurements and able to predict sex from nestling measurement data to 83% accuracy [18]. Measurements of weight; wing, tail and tarsus length; and beak length and width were taken from chicks when leg bands were fitted prior to fledging, approximately 38 to 48 days after hatching. Males tend to be larger, however there is an overlap with females, and smaller males are often within the range of females [20]. Sex is only confirmed based on observation of courtship and breeding behavior or post-mortem. The estimated sex was used for the 12 sap-feeding individuals with fledgling data for whom sex was not known.

## 3. Results

### 3.1. Sap Feeding Observations

Forty-one observations of sap feeding were made, totaling 555 minutes of sap feeding observation. Twenty-one individual kākā were identified foraging for sap, the majority of which were observed once (Table 1). Sap feeding was observed on 28 occasions at the Wellington Botanic Garden during systematic behavioral sampling. Remaining observations of sap feeding were made at Karori Cemetery (Figure 1, 1 observation), Karori Park (1), KWS (5) and in the suburbs of Highbury (1) and Northland (5).

All observations of sap feeding at Wellington Botanic Garden were on *Eucalyptus leucoxylon*. Elsewhere in Wellington, sap feeding was observed on macrocarpa (*Cupressus macrocarpa*), Lawson cypress (*Chamaecyparis lawsoniana*), Himalayan cedar (*Cedrus deodara*) and mahoe (*Melicytus ramiflorus*).

**Table 1:** Number and total duration of behavioral observations of kākā observed foraging for sap in Wellington between November 2011 and May 2013.

Leg band code ^1^	Sap feeding observations			Season fledged	Sex	Method of sex determination ^3^
	No	Duration (mins)	Location ^2^			
BG-V	1	11	WBG	11/12	M	Estimate from DFA
B-KB	1	16	BG, KWS	08/09	F	Breeding
BP-P	1	8	KWS	02/03	M	Breeding
GG-V	6	122	Northland, BG	11/12	F	Estimate from DFA
GW-V	2	4	WBG	11/12	M	Estimate from DFA
KO-G	1	30	KWS	04/05	F	Breeding
LW-O	2	36	WBG	10/11	M	Estimate from DFA
MG-V	1	11	WBG	11/12	F	Estimate from DFA
MP-V	1	2	WBG	11/12	F	Estimate from DFA
MR-O	1	15	WBG	10/11	F	Breeding
MW-O	1	2	WBG	10/11	F	Estimate from DFA
O-MY	1	19	WBG	10/11	M	Estimate from behavior
PB-V	1	2	WBG	11/12	M	Estimate from DFA
PL-O	1	28	KWS	10/11	F	Estimate from DFA
RP-V	1	1	WBG	11/12	M	Estimate from DFA
V-YW	1	14	WBG	11/12	F	Breeding
WB-W	1	13	KWS	07/08	F	Estimate from DFA
WK-O	1	15	Highbury	10/11	F	Breeding
W-OO	1	52	Karori Park	08/09	F?	Unknown
YK-O	1	5	WBG	10/11	M	Estimate from DFA
Y-RO	2	53	Northland	08/09	F	Breeding
Unbanded	8	62	WBG, Karori Cemetery			
Unidentified	4	34	WBG			

^1^ Codes used by Karori Wildlife Sanctuary to describe colored leg band combinations. Birds are assigned a unique combination of three colored bands that are read from bird’s left to right, top to bottom. B = Blue, G = Green, K = Black, L = Lime, M = Mauve, O = Orange, P = Pink, R = Red, S = Silver, V = Lavender, W = White, Y = Yellow; ^2^ WBG = Wellington Botanic Garden, KWS = Zealandia-Karori Wildlife Sanctuary; ^3^ The sex of birds without confirmed sex based on breeding or behavioral observation was estimated using a discriminant function analysis based on nestling measurements.

**Figure 2:**
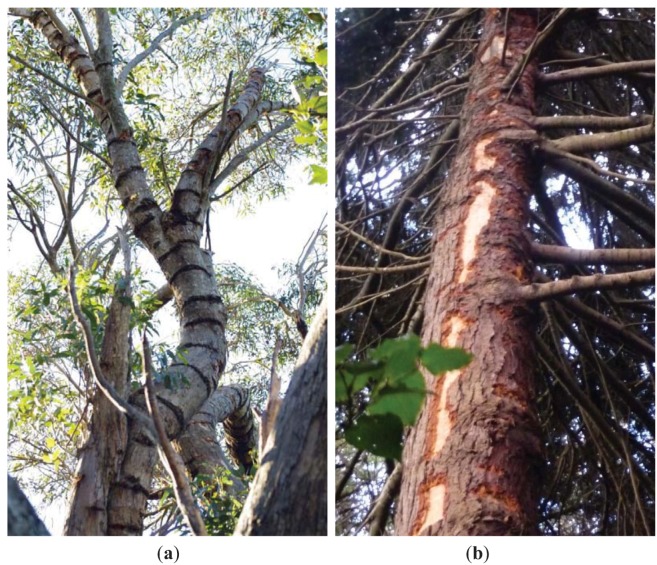
The two types of bark damage resulting from kākā sap feeding in Wellington, (**a**) transverse gouges and (**b**) removed patches of bark.

**Figure 3:**
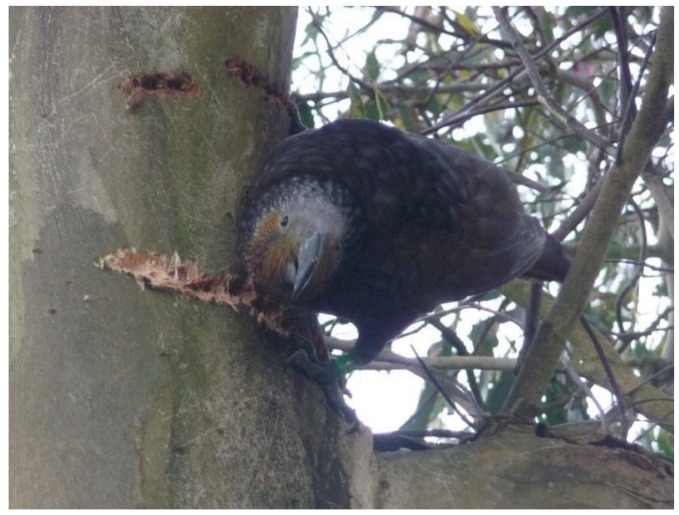
A juvenile kākā sap feeding by forming transverse gouges through the bark of *Eucalyptus leucoxylon*. Anderson Park, Wellington Botanic Garden, September 2012.

Two types of tree damage have been observed in Wellington; transverse gouges and removed patches of bark (Figure 2). Both adult and juvenile kākā were observed engaging in both types of bark damage and bark removal behavior differed between the two types of damage. Transverse gouges were made by using the beak to prise a deep gouge through the bark layer (Figure 3). The lower mandible was hooked under the bark and prised away from the bird, with the upper mandible held against the outer side of the bark to assist with leverage. This resulted in the bark gouge being extended away from the bird. Lapping sap and deepening or lengthening the gouge could not be distinguished as they occurred simultaneously. A sap foraging kākā would move between multiple gouges regularly during a sap feeding bout, spending between 1 second and 2.5 minutes at a single gouge (*x* ± SD, 45 ± 43 seconds, n = 21) before moving to another one nearby. Up to six bark gouges were utilized during a single sap feeding bout.

The other type of tree damage was characterized by the removal of patches of bark from a trunk or branch. The sap feeding bird would begin by prising off small pieces of bark until the cambial layer was reached. The mandible was then used to lever off small pieces of bark from around the edge of the bark wound, gradually enlarging its size. Bark was removed using the same technique as for horizontal gouges, with the upper mandible providing leverage and the lower mandible hooking under the bark and levering away from the bird. Removed pieces of bark were dropped to the ground often with a flicking motion of the head and the ground beneath recently damaged trees was often littered with bark pieces. At these types of bark wounds, a kākā would usually forage from only one or a few patches, alternating between enlarging the patch and lapping sap from edges that had previously been exposed. During this type of bark removal, lapping of sap could often be observed directly, particularly when large areas of cambium had been exposed. This occurred by the kākā holding its bill open and using the tongue to lick the exposed cambial surface, predominantly at the edges of the removed bark. The bird would also sometimes tilt its head and run the side of its bill over the exposed cambial surface lapping sap that had exuded. Sap was also often licked from the underside of a piece of bark immediately after it was removed from the tree. 

Despite their strong beak, removing bark took considerable time and effort. When initiating a bark wound a kākā may test various points on the bark, presumably to find a weak spot such as a knot or ridge in the bark. Once the kākā had a firm grasp under the bark, it would lever the bark off. The head and neck would be used to lever off thinner bark but for thicker barked trees, a kākā would use its entire body as leverage. Sometimes a kākā would move around to find an improved foothold to increase leverage. The sap feeding bird may stand on a lateral branch but often clung to the trunk in any orientation. 

The number of minutes spent sap feeding in a single bout ranged from less than 1 minute to 52 minutes, with a mean of 13.5 ± 12.5 minutes. Kākā were already engaged in sap foraging when sampling began in 34% (n = 14) of observations so bout length is likely to be underestimated. 

Sap feeding was observed on trees with many hundreds of scars and also on trees with no previous damage. Kākā returned to individual sap feeding trees repeatedly on multiple days for at least three weeks. Multiple observations of the removal of bark patches on three trees at two sites suggest that kākā may remove bark patches vertically down a tree trunk. Over the course of two to three weeks, new patches were observed to be removed beneath those already present or current patches were extended downward. No patterns were observed in the placement of transverse gouges on a tree. 

All bark removal observed during this study was assessed to be for the purpose of sap foraging. Invertebrate feeding was not observed during the study, however invertebrates may have been gleaned while removing bark to access sap. 

### 3.2. Characteristics of Sap Feeding Individuals

Eight kākā observed sap feeding were identified as juveniles (38% of identified sap feeding kākā), having fledged in the 2011/2012 breeding season. Seven kākā fledged in 2010/2011, three in 2008/2009, and one each in 2007/2008, 2004/2005 and 2002/2003 (Table 1).

Six individuals observed sap feeding were known from previous breeding history to be females and one was known to be male (Table 1). Behavioral observation of attempted mating during the course of this study suggested that one other bird observed sap feeding was male. The sex of one sap feeder was unknown as it was found as a juvenile, so nestling measurements were not obtained. The sex of 12 of the remaining 13 identified birds was estimated from nestling measurements. Six were estimated to be male and six female. Based on individuals whose sex was known or able to be estimated (n = 20, *i.e.*, excluding one banded bird), 60% of sap feeding birds were female (n = 12). This is not significantly more than expected by chance (exact binomial test, *p* = 0.868).

Sap feeding appears to be a predominantly solitary activity. Of the 41 observations of sap feeding, only four occurred when a conspecific was in the same tree and two with a conspecific within 1 meter of the sap feeder. Vocalization was rare during sap feeding, compared to frequent calling during other foraging behaviors. Sap feeding bouts were significantly longer when there were no conspecifics present in the area (no conspecifics observed or heard from the observation point, unequal variances *t*-test, t_15_ = 4.96, *p* < 0.001). The mean length of a sap foraging bout was 26.3 ± 12.8 minutes (n = 13) when the feeder was alone and 7.6 ± 6.6 minutes (n = 28) when conspecifics were present in the area.

We recorded eight instances of a kākā displacing a conspecific from a bark wound. This usually occurred simply by the bird approaching the sap feeding kākā who then flew off or moved away. The displaced kākā usually resumed sap feeding at a nearby wound within one minute. We observed one instance of a sap-feeding kākā chasing away a conspecific that landed nearby and two instances where a newly arrived bird unsuccessfully attempted to displace a sap feeding bird from a bark wound. On one occasion a tui (endemic honeyeater, *Prosthemadera novaeseelandiae*) was observed attempting to access sap while a kākā was foraging at bark wounds on Lawson cypress. The tui approached a recently made bark wound and was chased away by the kākā. After three similar attempts, the tui was able to access a bark wound and fed on sap from the edges of the removed bark for four minutes before being chased away again by the kākā. 

Twenty of the twenty-one individuals observed sap feeding during this study have been recorded during monthly surveys or by a microchip reader as visiting supplementary feeding stations within KWS in 2012. These birds were recorded feeding from stations between 1 and 1,696 times over the course of the year. Two of the females observed feeding on sap bred at monitored nests within Karori Wildlife Sanctuary in the 2012/13 season and an additional two birds bred in both the 2011/12 and 2012/13 seasons. The known male bred in the season preceding his observation.

## 4. Discussion

Although a small sample, the 41 observations of sap feeding and 21 sap feeding kākā individually identified during this study are a considerable increase on previous studies. 

Sap feeding was observed in kākā ranging in age from approximately six months to ten years old. Thirty-eight percent of identified sap-feeding birds were juveniles and 71% were less than 2 years old. Hence it can be concluded that sap feeding is not restricted to adults. Contrary to the suggestion of Berry [14], the complexity or skills required for either type of sap foraging does not restrict this technique to older birds. Young birds appeared to be competent at removing bark and foraging for sap. Therefore, if sap foraging is a learned behavior, young kākā are able to quickly master the technique during their first months after fledgling. The high proportion of young birds observed sap feeding may suggest that sap feeding occurs predominantly in younger birds. However, the demography of the Wellington City population is highly skewed due to recent expansion so this bias more likely reflects the higher proportion of young individuals in the population. 

Only one confirmed male was observed sap feeding. However, an additional six sap feeding individuals were estimated to be male based on body measurements as fledglings and another individual displayed male mating behavior. Only one other study has reported a male kākā sap feeding [9] and it has been suggested that sap feeding is predominantly a female behavior [9,14,21]. The results of this study do not clarify whether males seldom feed on sap or whether the few observations of males in this and previous studies are due to small sample sizes. Future behavioral and breeding observations may enable confirmation of the sex of the birds observed sap feeding during this study. 

The high proportion of juveniles coupled with the observation of confirmed and estimated males sap feeding suggests that sap is not only a supplementary food for females before breeding, but is important across the lifecycle for both sexes. Therefore, tree damage from sap feeding may also occur in other New Zealand cities where kākā are resident but do not breed or where they are occasional visitors. 

The observation of tui feeding on sap during this study is the first report of another species feeding on sap from a tree wounded by kākā. Exploiting sap from the bark wounds of other sap feeding species is common and, in Australia, sap availability has been found to have a strong effect on community structure [22]. It is possible that other New Zealand bird species may also make use of this resource and sap may provide a valuable resource for birds living sympatrically with kākā.

We did not find evidence that kākā that utilized supplementary food fed less on sap. The majority of birds observed sap feeding during this study (20 of 21 birds) accessed supplementary food at feeding stations within KWS during 2012, and supplementary food provisioning is high among Wellington residents [12]. This suggests that sap feeding in Wellington is not a response to food limitation and is unlikely to be necessary to meet energy needs. Converse to suggestions of Beaven [9] and Innes [17], further provision of supplementary food is unlikely to be an effective strategy for reducing sap foraging damage. 

Kākā move between food sources over the seasonal cycle [23], and sap-foraging is likely to vary across the year. Since the majority of observations for this study took place in late winter and early spring, additional observations at other times of year may provide further insight into sap-foraging behavior. 

It is possible that sap may contain signals for breeding or nutrients not available in other foods [9,16]. Other parrot species such as sulphur-crested cockatoo (*Cacatua galerita*) and little corella (*Cacatua sanguinea*) chew wood to assist in beak maintenance [24,25], and bark removal may have a similar function for kākā. Bark removal may also play a role in beak development for juvenile kākā. These hypotheses could be tested by nutrient analyses of sap and experiments with captive or free-ranging kākā.

Australian native parrots, such as the sulphur-crested cockatoo, cause similar problems in urban landscapes to those caused by kākā [24]. Particularly when attracted by supplementary food provision, these birds damage wiring, buildings, garden furniture and solar water heating systems on rooftops [24,25]. A range of strategies have been implemented in an attempt to reduce damage, including the use of chemical deterrents; scaring methods, such as noise and decoys; and physical exclusion using netting, wire and spikes [25,26]. However, these techniques have had limited success and in many cases population control has been required to mitigate problems [25]. The Australian experience demonstrates the potential for intelligent parrots to generate serious and intractable conflict in urban areas, and highlights the importance of understanding and managing urban wildlife issues proactively. 

## 5. Conclusions

The time and energy expended to access sap by kākā of both sexes and across all life stages, as well as the defense of sap feeding scars from conspecifics and other species, suggests that sap is a highly valued resource. Although further research to understand the reasons for sap feeding are warranted, it appears unlikely that kākā sap feeding in Wellington City can be reduced unless the kākā population is controlled. 

Management of the emerging conflict with Wellington residents should focus on reducing the negative impacts of the bark damage that results from sap feeding. This may include monitoring potentially hazardous trees with high levels of damage to mitigate risks to people, retaining trees that are favored by kākā for sap feeding in areas where they pose minimal hazard, and increasing the proportion of damage tolerant species in the landscape by planting species with greater tolerance to bark damage on public and private land. 

Although understanding the ecology and behavior of a conflict species is important to inform conflict management [27], successful mitigation of human-wildlife conflict requires an integrated understanding of the ecological and social dimensions of the problem [28]. Since the level of conflict is rarely proportional to the degree of damage, and urban perceptions of wildlife are often based on emotional and symbolic values, urban wildlife conflict can seldom be mitigated solely by reducing the impact of wildlife [28,29,30]. Hence, social research would be valuable to investigate Wellington residents’ attitudes to kākā and their experience of and tolerance towards damage. Social research will give an understanding of the extent and magnitude of conflict in Wellington City and may lead to social approaches to conflict mitigation.
